# Supplementary material for: Relationship of Anthropometric Indicators of General and Abdominal Obesity with Hypertension and Their Predictive Performance among Albanians: A Nationwide Cross-Sectional Study
Source: Nutrients. 2021 Sep 25;13(10):3373. doi: 10.3390/nu13103373 (PMC8537494; doi:10.3390/nu13103373)
Supplement: Supplementary file 1 [file nutrients-13-03373-s001.zip › nutrients-1369315-supplementary.pdf]

Supplementary material

Figure S1

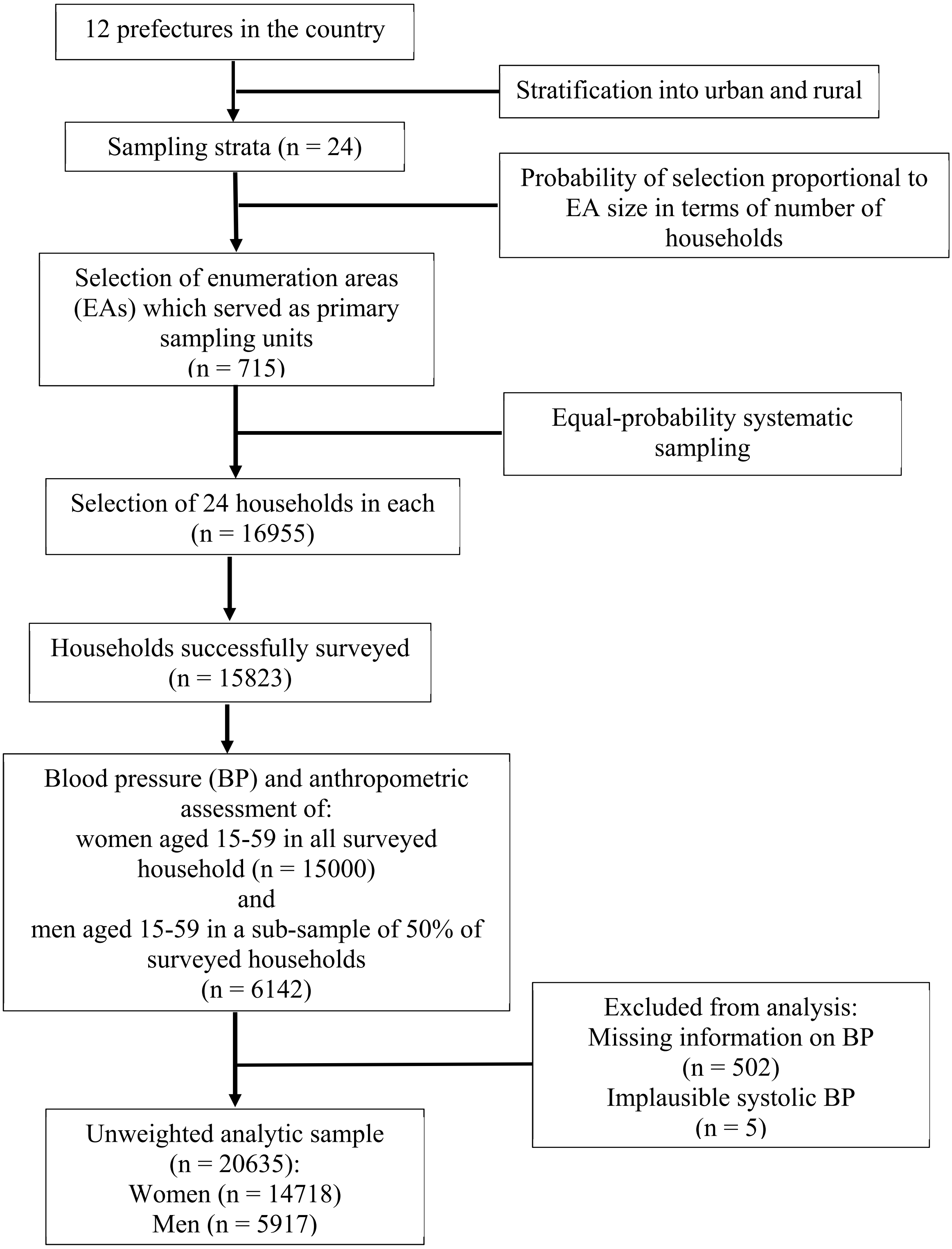

Figure S1. Flowchart illustrating inclusion of participants into the present study.

Figure S2

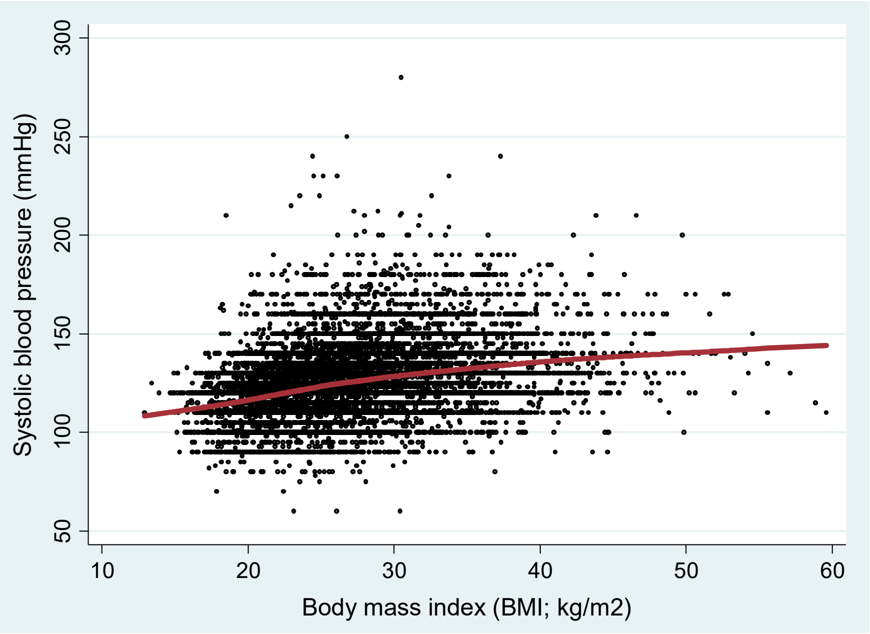
Systolic blood pressure (mmHg)
50
100
150
200
250
300
10
20
30
40
50
60
Body mass index (BMI; kg/m2)

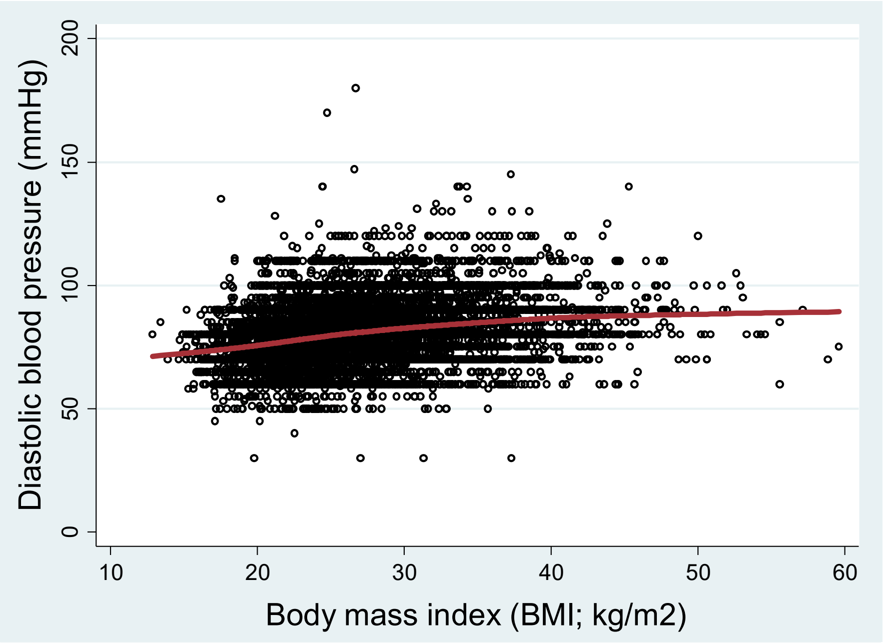
Diastolic blood pressure (mmHg)
0
50
100
150
200
10
20
30
40
50
60
Body mass index (BMI; kg/m2)

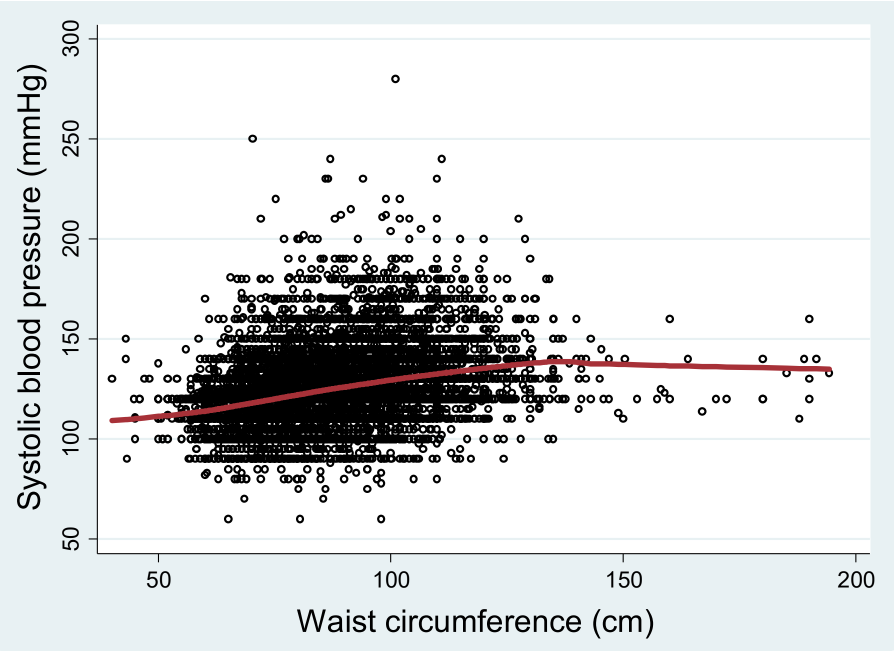

Systolic blood pressure (mmHg)
50
100
150
200
250
300
50
100
150
200
Waist circumference (cm)

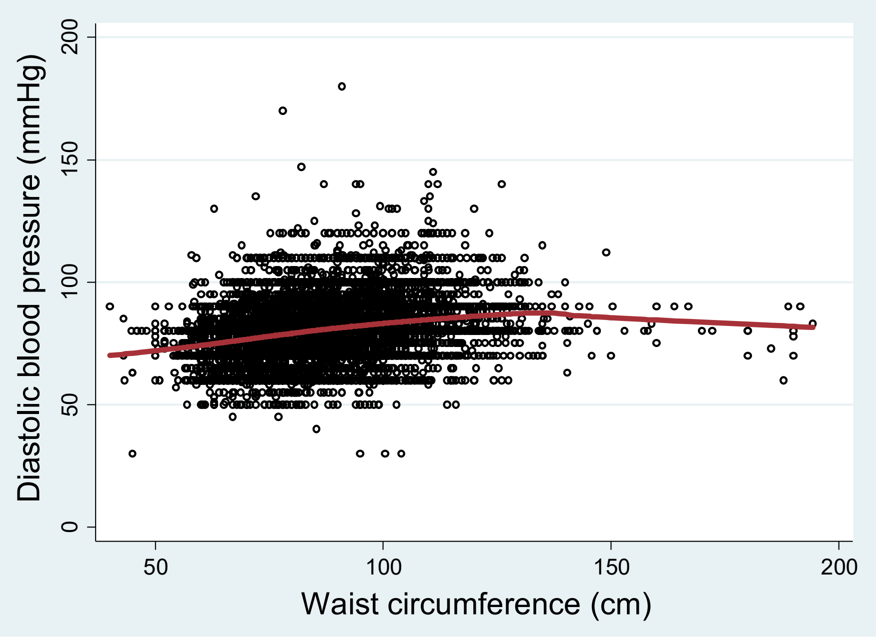

Diastolic blood pressure (mmHg)
0
50
100
150
200
50
100
150
200
Waist circumference (cm)

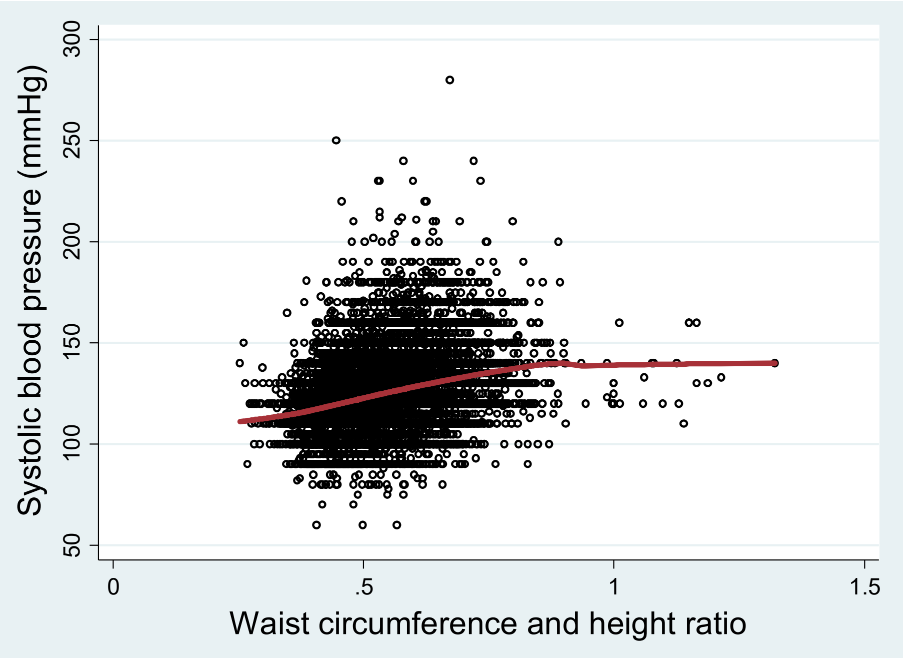

Systolic blood pressure (mmHg)
300
250
200
150
100
50
0
.5
1
1.5
Waist circumference and height ratio

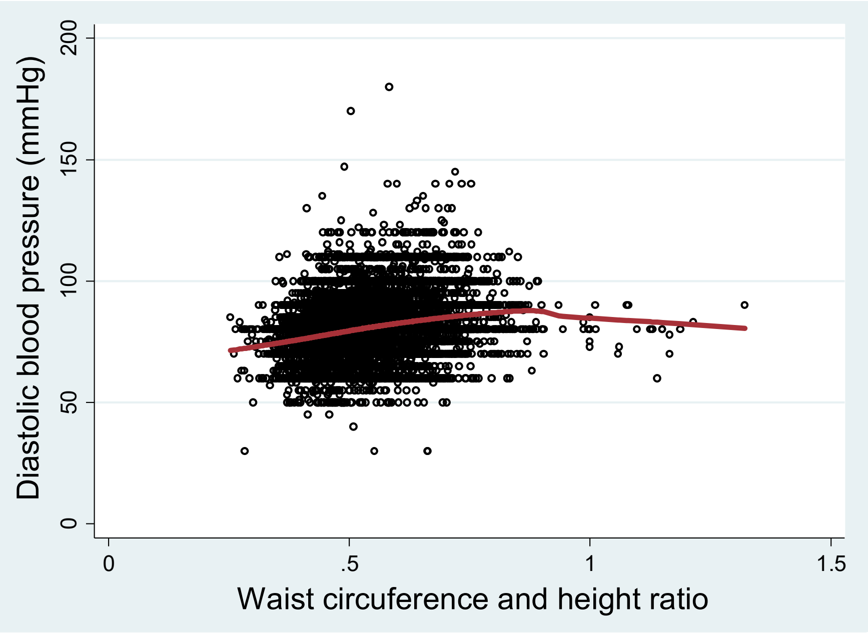

Diastolic blood pressure (mmHg)
200
150
100
50
0
0
.5
1
1.5
Waist circuference and height ratio

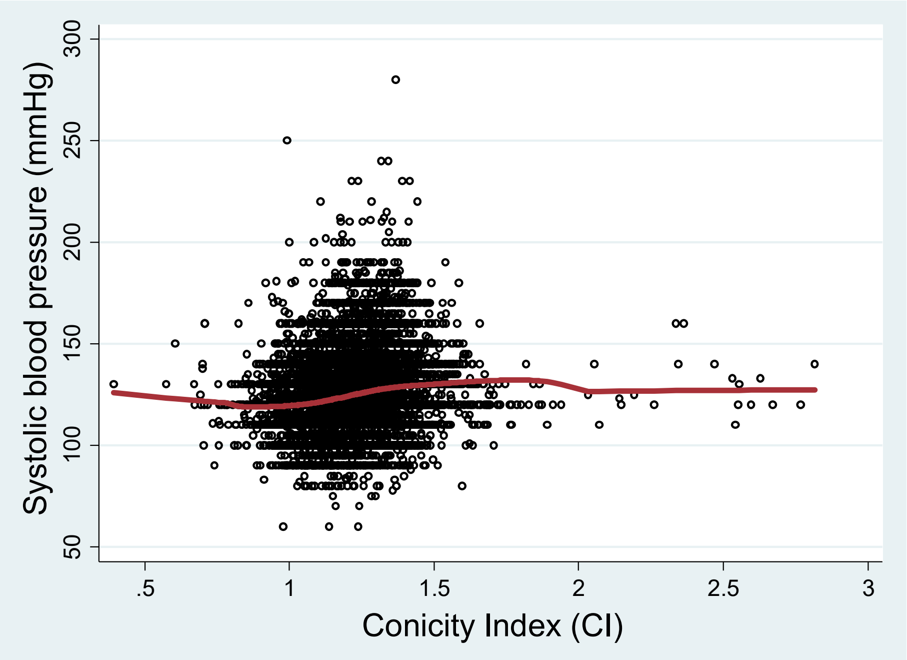

Systolic blood pressure (mmHg)
50
100
150
200
250
300
.5
1
1.5
2
2.5
3
Conicity Index (CI)

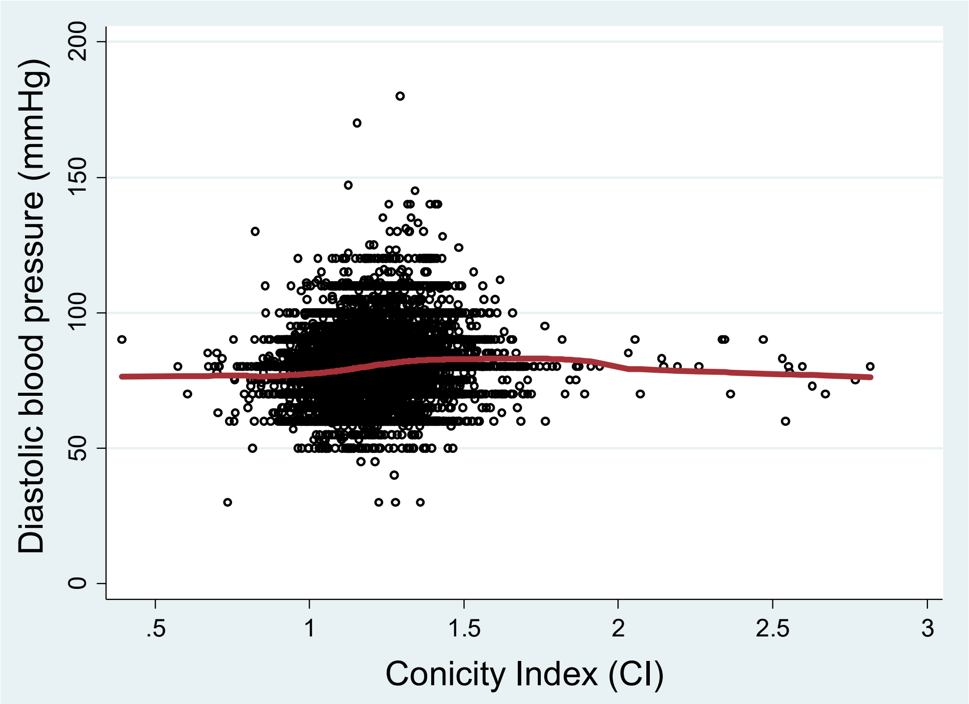

Diastolic blood pressure (mmHg)
0
50
100
150
200
.5
1
1.5
2
2.5
3
Conicity Index (CI)

Figure S2. Scatterplots showing systolic and diastolic blood pressures plotted against the four anthropometric indicators along with LOWESS (Locally Weighted Scatterplot Smoothing) lines.

Figure S3

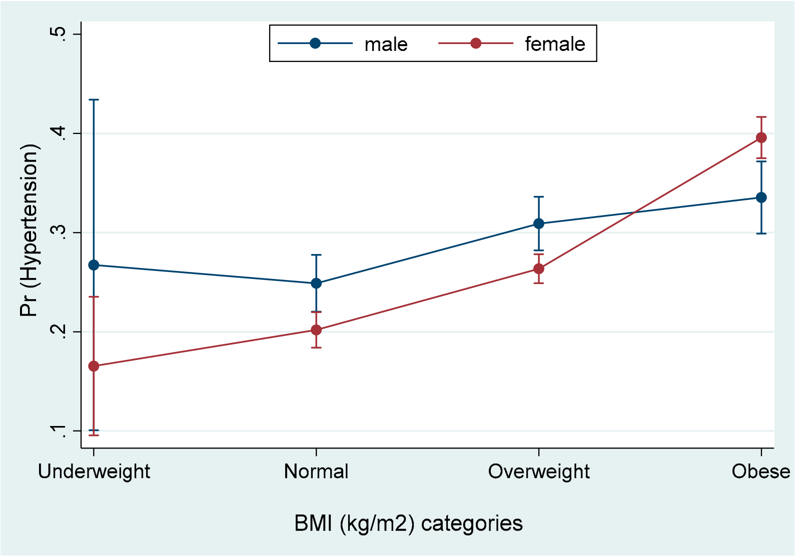

Predictive Margins of HTN probability for BMI (kg/m^2) categories with 95% CIs by sex

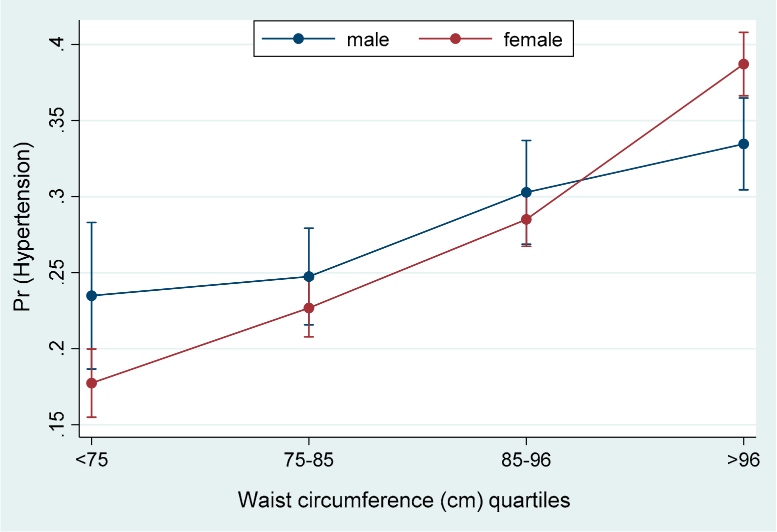

Predictive Margins of HTN probability for waist circumference (cm) quartiles with 95% CIs by sex

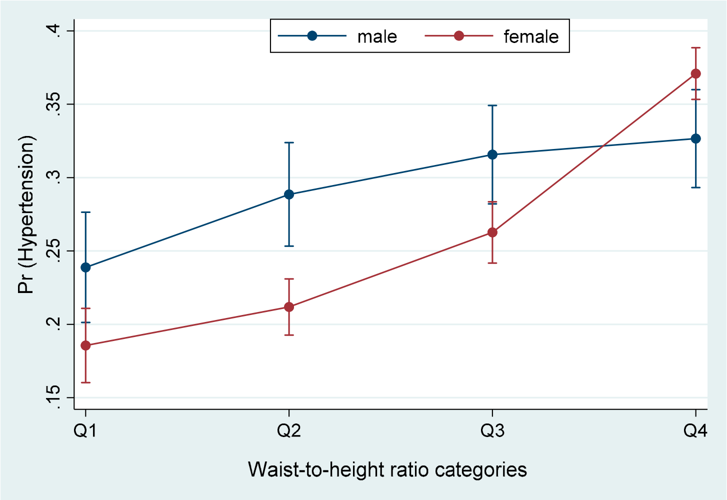

Predictive Margins of HTN probability for waist-to-height ratio categories with 95% CIs by sex

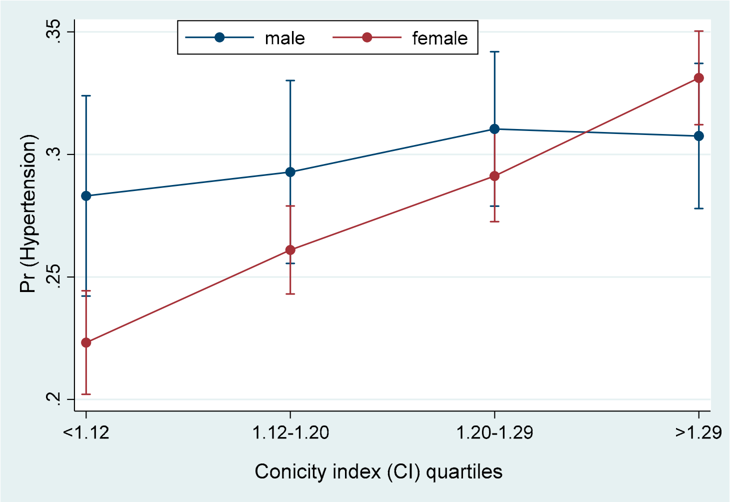

Predictive Margins of HTN probability for Conicity index (CI) quartiles with 95% CIs by sex

Figure S3. Plots of predictive margins with corresponding 95% confidence intervals for each category of the four anthropometric indicators by gender. In the highest category for all four indicators, the red lines representing the females surpassed the blue lines representing the males.
